# Comprehensive analysis of the transcriptional landscape of the human *FMR1* gene reveals two new long noncoding RNAs differentially expressed in Fragile X syndrome and Fragile X-associated tremor/ataxia syndrome

**DOI:** 10.1007/s00439-013-1356-6

**Published:** 2013-09-05

**Authors:** Chiara Pastori, Veronica J. Peschansky, Deborah Barbouth, Arpit Mehta, Jose P. Silva, Claes Wahlestedt

**Affiliations:** 1 Department of Psychiatry and Behavioral Sciences and Center for Therapeutic Innovation, Hussman Institute for Human Genomics, University of Miami, Miller School of Medicine, Miami, FL 33136 USA; 2 Division of Clinical and Translational Genetics, Dr. John T. Macdonald Foundation Department of Human Genetics, University of Miami, Miller School of Medicine, Miami, FL 33136 USA; 3 Hussman Institute for Human Genomics, University of Miami, Miller School of Medicine, Miami, FL 33136 USA

## Abstract

The majority of the human genome is transcribed but not translated, giving rise to noncoding RNAs (ncRNAs), including long ncRNAs (lncRNAs, >200 nt) that perform a wide range of functions in gene regulation. The Fragile X mental retardation 1 (*FMR1*) gene is a microsatellite locus that in the general population contains <55 CGG repeats in its 5′-untranslated region. Expansion of this repeat region to a size of 55-200 CGG repeats, known as premutation, is associated with Fragile X tremor and ataxia syndrome (FXTAS). Further expansion beyond 200 CGG repeats, or full mutation, leads to *FMR1* gene silencing and results in Fragile X syndrome (FXS). Using a novel technology called “Deep-RACE”, which combines rapid amplification of cDNA ends (RACE) with next generation sequencing, we systematically interrogated the *FMR1* gene locus for the occurrence of novel lncRNAs. We discovered two transcripts, *FMR5* and *FMR6*. *FMR5* is a sense lncRNA transcribed upstream of the *FMR1* promoter, whereas *FMR6* is an antisense transcript overlapping the 3′ region of *FMR1*. *FMR5* was expressed in several human brain regions from unaffected individuals and from full and premutation patients. *FMR6* was silenced in full mutation and, unexpectedly, in premutation carriers suggesting abnormal transcription and/or chromatin remodeling prior to transition to the full mutation. These lncRNAs may thus be useful as biomarkers, allowing for early detection and therapeutic intervention in FXS and FXTAS. Finally we show that *FMR5* and *FMR6* are expressed in peripheral blood leukocytes and propose future studies that correlate lncRNA expression with clinical outcomes.

**Electronic supplementary material:**

The online version of this article (doi:10.1007/s00439-013-1356-6) contains supplementary material, which is available to authorized users.

## Introduction

Trinucleotide repeat expansions give rise to more than 30 neurological and neuromuscular diseases, including Huntington’s disease (HD), Fragile X Syndrome (FXS) and Spinocerebellar Ataxia (Lopez Castel et al. 2010; Mirkin 2007). FXS, an X-linked genetic disorder, is the leading cause of inherited intellectual disability, and is otherwise characterized by behavioral problems and specific physical dysmorphisms. It is caused by an expansion of CGG repeats in the 5′ untranslated region (5′-UTR) of the Fragile X mental retardation 1 gene (*FMR1*). The 5′-UTR of *FMR1* contains 6–54 repeats in the general population; however, this region occasionally expands in subsequent generations. A size of 55–200 repeats is called a “premutation” (PM), while an expansion beyond 200 repeats is called a full mutation and results in Fragile X Syndrome in males. In most patients with FXS, both an upstream CpG island and the expanded CGG/CCG repeats are hypermethylated (Hornstra et al. 1993; Sutcliffe et al. 1992). This hypermethylation is associated with hypoacetylation of histones H3 and H4 in the promoter and the 5′ UTR of *FMR1* (Coffee et al. 2002; Coffee et al. 1999), leading to chromatin condensation and transcriptional silencing. The protein encoded by FMR1, FMRP, is an RNA-binding protein involved in translational repression, synaptic maturation, dendritic mRNA localization and nucleoplasmic shuttling of mRNA (Antar et al. 2005; Brown et al. 1998; Weiler et al. 1997) lending support to the idea that its aberrant expression contributes to the intellectual disabilities (ID) associated with FXS (Hinton et al. 1991; Irwin et al. 2000; O’Donnell and Warren, 2002). FMRP may also have other important functions in the nucleus, according to a recent study showing that FMRP binds methylated H3K79 chromatin and mediates the DNA-damage response pathway (Shi et al. 2012). *FMR1* premutation carriers are at risk for developing FXTAS, a neurodegenerative condition affecting approximately 46 % of males and 17 % of females (Garcia-Arocena and Hagerman 2010). FXTAS is characterized by ataxia, parkinsonism, intentional tremors, psychiatric symptoms and cognitive decline with onset usually after 50 years of age (Hagerman et al. 2001). Additionally, approximately 20 % of female premutation carriers are at an increased risk for FXPOI (Allingham-Hawkins et al. 1999). Although each Fragile X-associated condition does have a characteristic phenotype, great variability exists in severity and penetrance.

Furthermore, the molecular mechanisms by which FXS and FXTAS/FXPOI arise are largely unrelated. In FXS, the symptoms are due to the silencing of *FMR1* and ensuing lack of FMRP, while in FXTAS/FXPOI the expansion does not silence the *FMR1* gene. In fact, FMRP levels in FXTAS are not or only slightly reduced compared to the normal population whereas *FMR1* mRNA expression levels are increased two to eightfold, suggesting an RNA toxicity mechanism underlying this condition. Although the precise mechanism for this overexpression is still unknown, it has been postulated that a longer tract of CGG repeats near the *FMR1* promoter results in a more open chromatin state, thereby promoting access by transcription factors (Kenneson et al. 2001; Tassone et al. 2000).

Vast genomic regions are transcribed but not translated and many of the resulting transcripts, known as noncoding RNAs (ncRNAs), are enriched in the brain (Banfai et al. 2012; Cheng et al. 2005; Djebali et al. 2012). Long ncRNAs (lncRNAs), which are ncRNAs longer than 200 nucleotides, perform a wide range of functions, including modulation of transcription or of the epigenetic landscape of their loci of origin. LncRNAs can be transcribed from the sense and antisense strands of protein-coding genes, and can arise from introns, promoters and 3′ end regions (Djebali et al. 2012; Mattick, 2005; Yan and Ma, 2012). Transcriptomic studies have revealed that antisense transcription is a common feature of mammalian genes that are actively transcribed from microsatellite disease loci (Cho et al. 2005; Ladd et al. 2007; Moseley et al. 2006). Furthermore, others and we have recently reported that lncRNAs emanate from the *FMR1* gene locus and are differentially expressed in both FXS and premutation carriers (Khalil et al. 2008; Ladd et al. 2007). These *FMR1*-derived lncRNAs are primate-specific, and animal models have not addressed their potential influence on the FXS phenotype (The Dutch-Belgian Fragile X Consortium 1994; Chen and Toth 2001; Fisch et al. 1999; Godfraind et al. 1996; Miller et al. 1999; Stafstrom et al. 2012). It is possible that ncRNAs produced from the *FMR1* locus may modulate certain aspects of FXS/FXTAS as has been demonstrated in other human diseases [reviewed in (Pastori and Wahlestedt 2012)]. We hypothesize that ncRNA may contribute to and be reflective of clinical variability in humans and hence could be used as biomarkers for FXS/FXTAS.

Here, we employed a recently developed method called Deep-RACE (Olivarius et al. 2009) to comprehensively search the entire *FMR1* locus for novel lncRNAs. The NCBI database reports several antisense-oriented ESTs (Expressed Sequence Tags) mapping to the *FMR1* locus, suggesting the presence of as yet uncharacterized transcripts. By performing rapid amplification of cDNA ends (RACE) on total human brain RNA followed by next generation sequencing, we have identified two new transcripts that we refer to as *FMR5* and *FMR6*. The expression of these newly described RNA species was validated in several regions of unaffected human brain tissue as well as in brain samples from FXS and premutation carriers. Our work provides a systematic analysis of the complex transcriptional landscape of the *FMR1* locus, uncovering two novel lncRNAs.

## Materials and methods

### Deep-RACE

Here we modified an existing protocol developed in 2009 to identify in a high-throughput manner the transcription start site of genes of interest using 5′RACE (Olivarius et al. 2009). We applied the same strategy using 3′RACE to detect the end of transcripts of interest.

### Sense oriented 5′ RACE

5′ RACE for sense-oriented novel transcripts was performed using the Invitrogen 5′ RACE System according to manufacturer instructions (Invitrogen cat#18374-058). Briefly, strand-specific cDNA was synthesized from 1 μg of total human brain RNA (Clontech, cat#636530) using gene-specific primers (GSP1, Online Resource 3) located 500 bp upstream the TSS of *FMR1*. After first strand cDNA synthesis, a homopolymeric tail was added to the 3′-end of the cDNA, using TdT enzyme and dCTP. Tailed cDNA was then amplified using gene-specific primer 2 (GSP2, Online Resource 3) and the Abridged Anchor Primer (AAP) provided with the system. PCR products were re-amplified in a nested PCR using gene-specific primer 3 (GSP3, Online Resource 3) and the abridged universal amplification primer (AUAP) provided by the kit. The final PCR products were submitted for next generation sequencing.

### Antisense oriented 5′RACE

5′ RACE for novel antisense transcripts was performed using the 5′ RACE System according to manufacturer instructions (Invitrogen cat#18374-058). Briefly, strand-specific cDNA was synthesized from 1 μg of total human brain RNA (Clontech) using gene-specific primers (GSP1, Online Resource 3) located in exon 1, exon 5 and exon 17, thereby spanning the entire locus of interest. cDNA was tailed with TdT enzyme and amplified using gene-specific primer 2 (GSP2, Online Resource 3) and AAP. PCR products were re-amplified with gene-specific primer 3 (GSP3, Online Resource 3) and AUAP. The final PCR products obtained from RACE experiments in the aforementioned regions of the locus were pooled and submitted for next generation sequencing.

### Sense oriented 3′RACE

The 3′RACE protocol is based on the concept that messenger RNAs are polyadenylated transcripts and can be converted, via the reverse transcription step, to cDNA using a 3′RACE adapter primer that binds the polyadenylated tail (polyA) of the RNA. Certain noncoding RNAs are polyadenylated while others are not, and the 3′RACE protocol (Ambion cat#AM1700) can only detect polyA transcripts. RNA that is not polyadenylated cannot be detected by this method.

Following reverse transcription, the cDNA is used in the following amplification steps: a first round of PCR was performed using the 3′RACE Outer primer (provided by the kit) and a gene-specific primer for the sense noncoding RNA located 1 kb upstream of the TSS (Online Resource 4). An additional PCR step was performed using the 3′RACE Inner primer and another gene specific primer. Nested PCR DNA products were submitted for next generation sequencing.

### Antisense oriented 3′RACE

Total Human Brain RNA was reverse transcribed to cDNA according to the manufacturer’s protocol as described above for 3′RACE-sense (Ambion cat#AM1700). The first round of PCR was performed using primers located in exon1, exon5 and exon17 in order to ensure coverage of the entire locus. Nested PCR was performed using inner gene specific primers located in the previously mentioned regions (Online Resource 4). PCR products from all regions of the locus were pooled and submitted for next generation sequencing.

### Next generation sequencing

The reads coming from sequencing of 5′- and 3′RACE PCR products from Hiseq2000 sequencer were prepared for Alignment by trimming the adapters from the beginning and the end of the reads using PERL programs. Mapping of the reads coming from the sequencing of 5′- and 3′RACE PCR products was conducted using version 2.0.1 of TopHat, using default settings for Illumina reads. All reads were aligned to the hg19 assembly version (GRCh37) of the human genome and the prebuilt index of the hg19 genome assembly (TopHat) was acquired from the TopHat homepage (http://tophat.cbcb.umd.edu/).

### Collection of tissue samples

19 human brain tissue samples were obtained from the NICHD Brain and Tissue Bank for Developmental Disorders at the University of Maryland, Baltimore, MD (Online Resource 5). RNA was extracted from tissue using the Trizol-Chloroform protocol and DNAse treated.

### RNA from patient brain tissue and RNA from patient blood

RNA from the cerebellum of 4 full mutation patients (MIND1031-09LZ, MIND1031-08GP, MIND1033-08WS, MINDJS-03) was provided courtesy of Dr. Tassone, UC Davis, MIND Institute, CA. Detailed information about patient’s samples can be found in Online Resource 5.

Blood from 2 control (616-11-ST, 378-11-JM), 2 premutation (288-12-JC, 453-12-EG) and 2 full mutation (22-12-FD, 294-12-LP) patients was provided courtesy of Dr. Tassone and it was processed to extract RNA (Tempus tubes, Applied Biosystems) according to University of California, Davis, Institutional Review Board-approved human subject protocols.

### CDNA synthesis and quantitative PCR

The two novel transcripts, *FMR5* and *FMR6*, were validated in several human brain regions. Strand specific reverse transcription (RT) was performed on 200 ng of commercial RNA (Clontech cat #636530, #636593, #636535, #636563, #636564, #636526, #636570, #636561) to make cDNA specific for *FMR5* and *FMR6*. To rule out DNA contamination in the RNA samples, we included a “No RT” condition, in which the reverse transcriptase enzyme was omitted from the reaction. The primers used in the RT are reported in Online Resource 6.

Quantitative PCR (qPCR) was used to compare the expression of *FMR5* and *FMR6* in commercially available RNA from several brain regions (Clontech), between human brain specimens and lymphocytes from control, premutation and full mutation individuals. *FMR5* was measured using a custom TaqMan probe while *FMR4* and *FMR6* were quantified using SYBR Green, and the primers used were validated by melting curve. Glucose-6-Phosphate Dehydrogenase and cyclophilin were used as housekeeping genes for expression normalization. QPCR data were analyzed by Delta Delta Ct method. Primers are listed in Online Resource 6.

## Results

The previous work reporting the presence of the antisense lncRNAs called *ASFMR1* and *FMR4* was performed in neuroblastoma (Khalil et al. 2008) and lymphoblastoid cell lines (Ladd et al. 2007), which display different epigenetic signatures than normal human brain potentially occluding discovery of additional transcripts. Here we attempted to identify novel transcripts derived from the *FMR1* locus in human brain from unaffected, FXS and FXTAS patients. We screened the *FMR1* locus for antisense transcripts by combining rapid amplification of cDNA ends (RACE) with next generation sequencing to determine the 5′ and 3′ends of novel transcripts. This technique, also called Deep-RACE, was also applied to search for sense-oriented lncRNAs upstream of the transcription start site (TSS) of *FMR1*. Sense oriented lncRNAs overlapping gene promoters (Han et al. 2007; Kurokawa 2011; Martianov et al. 2007; Song et al. 2012) have been shown to regulate transcription initiation (Martianov et al. 2007; Song et al. 2012) and may therefore contribute to *FMR1* gene dysregulation in FXS/FXTAS.

**Fig. 1:**
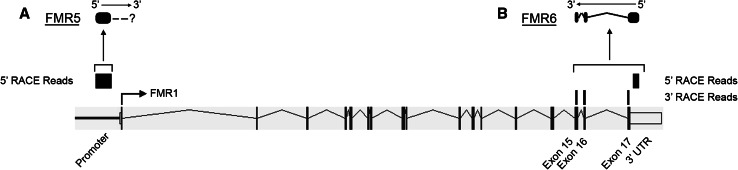
Alignment of Deep-RACE results to the *FMR1* locus. The reads obtained by Deep-RACE were visualized using the IGV (Integrative Genomic Viewer) program after their alignment to the human genome, and appear as *grey bars* above the schematic of the *FMR1* gene. **a** Those located in the promoter of *FMR1* are the result of the 5′RACE, and represent a partial sequence of *FMR5*, the sense transcript. **b** The reads that map to exon 15, exon 16, exon 17 and the 3′UTR of *FMR1* represent the antisense transcript, *FMR6*, and result from 5′ and 3′ RACE deep sequencing

We interrogated the entire *FMR1* locus by performing 5′- and 3′RACE spanning four different regions: the *FMR1* promoter (up to 1 kb from the TSS), exon 1, exon 5 and exon 17. Our choice to explore these regions was based on reported expressed sequence tags antisense to the *FMR1* locus. We confirmed RACE-PCR amplification of novel transcripts by capillary electrophoresis (Online Resource 1). These RACE PCR products were subjected to paired-end sequencing on Illumina’s HiSeq2000 sequencer. This procedure identified two novel noncoding RNAs, *FMR5* (GenBank KC894604) and *FMR6* (GenBank KC894603). *FMR5* is a sense-oriented, unspliced 800 nt long transcript whose 5′-end maps approximately 1 kb upstream of the TSS of *FMR1* (Fig. 1a, Online Resource 2). We were unable to obtain 3′RACE products for *FMR5*. One possible reason is that the high GC content in the promoter and 5′UTR of *FMR1* interfered with the RACE reaction. *FMR6* is a spliced 600 nt long antisense transcript whose sequence is entirely complementary to the 3′ region of *FMR1*. It begins in the 3′UTR and ends in exon 15 of *FMR1* (Fig. 1b, Online Resource 2). Interestingly, sequencing reads for *FMR6* align only to the exons of *FMR1*, indicating that the transcript is spliced and that *FMR6* and *FMR1* share the same splice junctions.

By definition, a noncoding RNA is a transcript that lacks an open reading frame (ORF) and is therefore not translated. In eukaryotes, protein-coding transcripts commonly contain an ORF >300 nucleotides (100 amino acids). We used the National Center for Biotechnology Information’s (NCBI’s) “ORF Finder” to determine ORFs in our two novel transcripts. *FMR6* was found to contain a few short ORFs (~130 nt) (Online Resource 3). *FMR5* contained a short ORF of 114nt and one of 459 nt potentially encoding a protein of 153 amino acids (Online Resource 3).

To explore the possibility that this hypothetical protein is functional, we performed homology searches using the NCBI tool BLASTP. This query detected no putative conserved domains in any of the available databases (nr, refseq_protein, swissprot, pat, pdb and env_nr). Lack of homologous proteins and protein domains suggests that this sequence is unlikely to encode a functional protein. As a complementary strategy, we analyzed its potential domain profile using the Conserved Domain Architecture Retrieval Tool (Geer et al. 2002). This search also resulted in no hits, further supporting the idea that the 459aa ORF does not encode a functional protein.

**Fig. 2:**
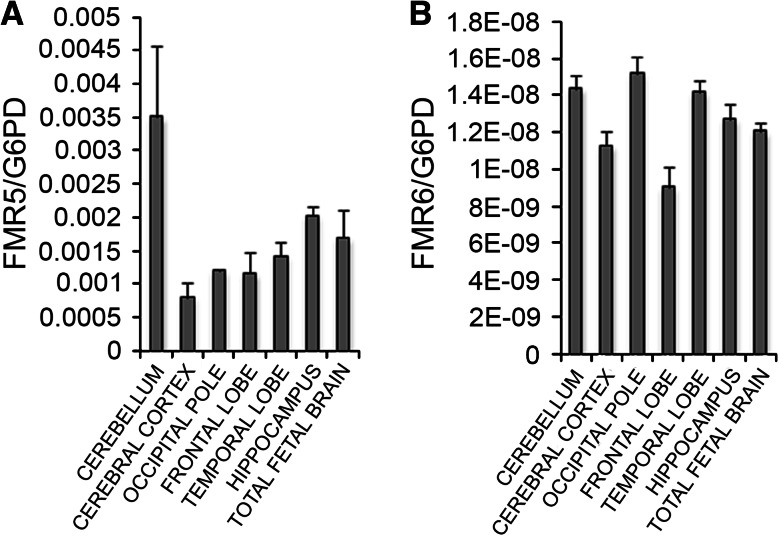
*FMR5* and *FMR6* are widely expressed in the human brain. Expression of *FMR5* (**a**) and *FMR6* (**b**) was measured in RNA from human cerebellum, cerebral cortex, occipital pole, frontal lobe, temporal lobe, hippocampus and total fetal brain with qPCR. Data were normalized to expression of the housekeeping gene, glucose-6-phosphate dehydrogenase (G6PD). *Error bars* represent the standard deviation of four technical replicates

We next assessed *FMR5* and *FMR6* expression in various human brain regions. *FMR5* and *FMR6* expression was detected at low levels in comparison to housekeeping genes in adult human cerebellum, frontal and temporal lobes, occipital and cerebral cortices and hippocampus, suggesting their presence in most adult brain regions (Fig. 2). The two transcripts were also expressed in fetal total brain RNA (Fig. 2); however, we do not yet have evidence for a developmental function of these transcripts.

**Fig. 3:**
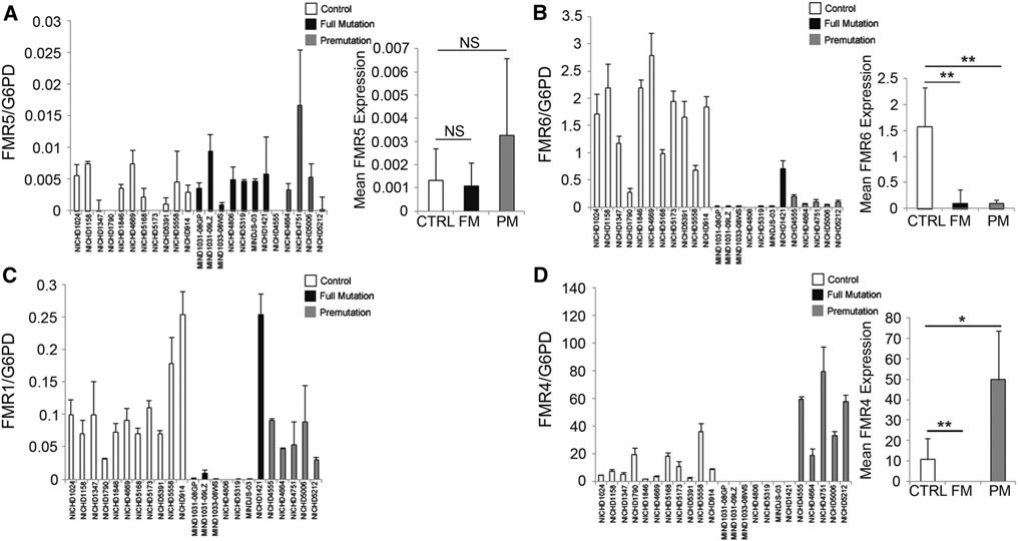
Expression of *FMR5*, *FMR6*, *FMR1* and *FMR4* in the brain tissue of control, full mutation and premutation individuals. Expression of *FMR5* (**a**), *FMR6* (**b**), *FMR1* (**c**) and *FMR4* (**d**) was measured in control, full mutation and premutation brain tissue by qPCR. Data were normalized to expression of glucose-6-phosphate dehydrogenase (G6PD), the housekeeping gene. In each panel, *error bars* for individual patients represent the standard deviation of four technical replicates. For mean expression values, *error bars* represent the standard deviation within control, FM and PM groups. *p < 0.05, **p < 0.01, *NS* not significant as determined by Student’s *t* test

To explore the possibility that these novel transcripts contribute to FXS pathogenesis, we assessed *FMR5* and *FMR6* expression in *post mortem* brain tissue from full and premutation individuals and unaffected controls. RNA expression was quantified in tissue from 11 controls of mixed gender, 7 male full mutation (FM) and 5 male premutation (PM) individuals. On average, *FMR5* expression did not differ significantly between full mutation, premutation and control samples (Fig. 3a). However, *FMR6* expression was significantly decreased in both the full mutation and premutation groups compared to controls, with the exception of sample #NICHD1421, which expresses *FMR1* at levels similar to controls (Fig. 3b). All other full mutation samples have, as expected, reduced expression of *FMR1* (Fig. 3c). We also determined expression levels of *FMR4*, a lncRNA previously reported by us (Khalil et al. 2008), in patient brain tissue. *FMR4* was not detectable in full mutation (FM) samples, and its expression was increased in premutation (PM) samples compared to controls (Fig. 3d). A no-RT negative control was included in the detection of all ncRNA transcripts to ensure that the signal was not accounted for by genomic DNA contamination. The levels of *FMR1*, *FMR4*, *FMR5* and *FMR6* were variable in FXS and premutation brain samples. It is likely that differences in the number of repeats, extent of DNA methylation or extent of histone modifications causes this variability. Variable expression levels of these transcripts may translate into different clinical outcomes. These lncRNAs may thus be useful as biomarkers for FXS and FXTAS. To determine whether lncRNA expression is linked to clinical phenotype, it is necessary to measure these transcripts in a large cohort of patients, and blood presents the most practical option for both experimental and potential prognostic purposes.

**Fig. 4:**
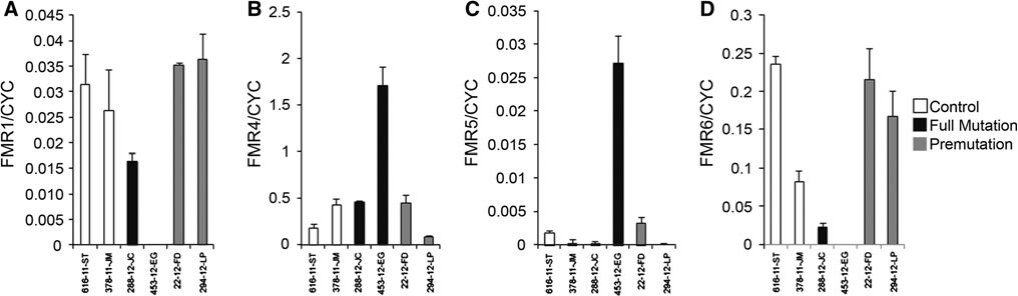
*FMR1, FMR4, FMR5 and FMR6* are detectable in peripheral blood leukocytes. Expression of *FMR1* (**a**), *FMR4* (**b**), *FMR5* (**c**) and *FMR6* (**d**) RNA was measured in leukocytes from the peripheral blood of control, full mutation and premutation individuals. Data were normalized to the housekeeping gene cyclophilin (CYC). *Error bars* represent the standard deviation of four technical replicates

To determine whether we can detect these novel lncRNAs in blood samples, we measured *FMR4*, *FMR5* and *FMR6* expression in RNA extracted from peripheral blood leukocytes in a limited number of patients (control *n* = 2, premutation *n* = 2, full mutation *n* = 2) by strand-specific reverse transcription quantitative PCR (RT-qPCR). All control and PM samples demonstrated robust *FMR1* expression; additionally, a moderate level of *FMR1* mRNA was detected in one of two FM patient samples, likely due to mosaicism (Fig. 4a). Expression of *FMR4*, *FMR5*, and *FMR6* was also detectable in the majority of patient leukocyte RNA samples albeit expression levels were lower than those of *FMR1* (Fig. 4b–d). These results establish the feasibility of conducting studies correlating expression levels of *FMR4*, *FMR5*, and *FMR6* with clinical outcomes in FXS and FXTAS patients.

## Discussion

**Fig. 5:**
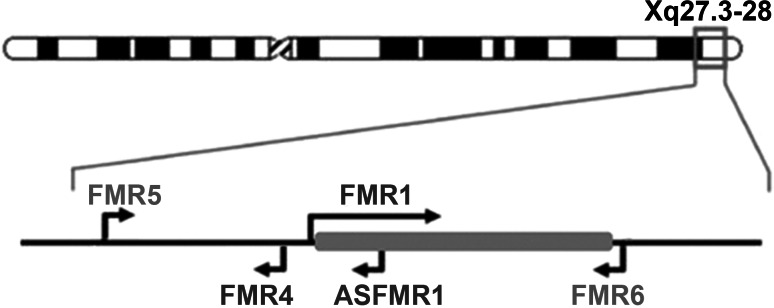
The transcriptional landscape of the *FMR1* gene locus is complex. Graphical representation of the previously described and newly reported lncRNAs expressed from the *FMR1* locus. *FMR5* is transcribed from the *FMR1* promoter in the sense direction, beginning around 1 kb upstream from the *FMR1* transcription start site. *FMR6* is an antisense-oriented lncRNA produced from the 3′UTR of *FMR1*

In recent years, the importance of lncRNAs in many aspects of cell biology has received increasing attention from the scientific community. For decades, these transcripts were regarded as transcriptional noise, and thus their role in many disease processes was overlooked. Growing evidence points to a multitude of functions performed by lncRNA, including regulation of transcription and chromatin remodeling (Lee 2012). Here we report the discovery of two novel transcripts that originate from the *FMR1* locus, adding complexity to a locus already known to produce other lncRNAs (Khalil et al. 2008; Kumari and Usdin 2010; Ladd et al. 2007) (Fig. 5).

The first lncRNA we report, *FMR5*, is a sense-oriented transcript that overlaps the *FMR1* promoter. The *FMR5* transcription start site (TSS) is located 1 kb upstream of the *FMR1* TSS. *FMR5* showed similar expression levels in control, FM and PM brain tissue, suggesting that FMR5 transcription remains independent of chromatin modifications in FM and PM carriers. This is consistent with the finding that in FXS repressive chromatin marks such as trimethylation of histone H3 at lysine 9 (H3K9me3) and trimethylation of histone H4 at lysine 20 (H4K20me3) associate with exon 1 of *FMR1*, which contains the CGG repeats, but do not associate with the promoter region (Kumari and Usdin 2010). On the other hand, levels of three active chromatin marks, H3 acetylation (H3Ac), H4 acetylation (H4Ac) and H3K4 dimethylation (H3K4me2), were reportedly lowered at the *FMR1* promoter in FXS (Gheldof et al. 2006). Furthermore, Kumari et al. (2010) reported the presence of uncharacterized antisense lncRNAs in the *FMR1* promoter of both normal and full mutation cells, suggesting that the presence of repressive histone marks in the *FMR1* locus would not necessarily inhibit the transcription of low abundance transcripts such as *FMR5*.

*FMR6* is a spliced antisense-oriented lncRNA that overlaps exons 15-17 as well as the 3′UTR of *FMR1.* Unexpectedly, the splicing sites in *FMR6* correspond exactly to those of *FMR1.* Although very little is known about the consensus sequences for splicing of noncoding RNAs, it is possible that the reverse complement of the canonical sites in *FMR1* are being recognized as non-canonical consensus sequences by the splicing machinery. Programs such as “Human Splicing Finder” (http://www.umd.be/HSF) can be used to predict non-canonical splicing sites by incorporating matrices for auxiliary sequences (Desmet et al. 2009). Further studies are required to address this possibility.

Our data show that *FMR6* expression is significantly downregulated in FXS brain samples, as is expected due to the reported decrease in H4Ac and H3K4me2 and increase in H3K9me2 throughout the *FMR1* locus, including the 3′ region of *FMR1* (Gheldof et al. 2006). However, DNA methylation is restricted to the CGG repeat region at 5′ end of the gene. One full mutation case in our study, NICHD#1421, displayed robust *FMR1* expression in addition to higher expression of *FMR6* compared to other FM patients. It is likely that in this case the 3′UTR is unaffected by the repressive chromatin modifications discussed above. Therefore it is possible that the observed reduction in *FMR6* expression is a consequence of histone changes associated with the FM, rather than the DNA methylation responsible for *FMR1* silencing.

An unanticipated result is that *FMR6* expression is reduced in premutation-range samples; however, the chromatin marks associated with the 3′ end of *FMR1* in premutation carriers are yet to be described. As mentioned previously, premutation-range expansions in the CGG repeat region are reported to result in an open chromatin state and increased *FMR1* transcription (Tan et al. 2009). Our data suggest that in addition, the premutation-range CGG expansion somehow influences transcription or chromatin state near the far-distal 3′ end of the gene at the premutation stage. Finally, we found that *FMR4*, similarly to *FMR1,* is downregulated in brain from FM patients and upregulated in PM carriers as we previously reported in blood leukocytes (Khalil et al. 2008).

As discussed above, *FMR5* and *FMR6* have distinct expression patterns, and additional studies are necessary to clarify whether any potential regulatory function of each transcript may contribute to FXS/FXTAS phenotypes. *FMR6* is complementary to the 3′ region of *FMR1* and may therefore bind to the *FMR1* mRNA, thereby regulating *FMR1*′s stability, splicing, subcellular localization and translational efficiency. These regulatory functions have been described for other lncRNAs (reviewed in (Faghihi and Wahlestedt 2009)). For instance, stability of *BACE1* mRNA is positively regulated by an antisense lncRNA to *BACE1* called *BACE1AS* (Faghihi et al. 2008). The possibility that *FMR6* regulates *FMR1* mRNA splicing may be relevant since extensive alternative splicing of *FMR1* has been demonstrated (Huang et al. 1996; Sittler et al. 1996). Finally, as *FMR6* overlaps two microRNA binding sites for miR-19a and miR-19b in the 3′UTR of *FMR1* (Edbauer et al. 2010), it is possible that this lncRNA can modulate stability or translational efficiency of *FMR1* through interference with microRNA binding.

The fact that *FMR1*-derived lncRNAs are differentially expressed in FXS and FXTAS suggests their usefulness as biomarkers for these diseases. The use of lncRNAs as biomarkers for human disease is a rather novel concept. LncRNAs have emerged as novel diagnostic/prognostic biomarkers in bodily fluid samples of cancer patients. One example is the lncRNA prostate cancer antigen 3, which can be detected in urine samples and has been shown to improve diagnosis of prostate cancer (de Kok et al. 2002; Reis and Verjovski-Almeida 2012). Investigating the relationship between differential lncRNA expression and clinical outcomes requires screening a large number of patients with various degrees of defined FXS or FXTAS symptoms. We have demonstrated through our six-patient pilot study that such a screening can be performed, as *FMR4*, *FMR5* and *FMR6* are detectable in peripheral blood leukocytes. If variability in expression of these transcripts in FM and PM individuals correlates with clinical variability, it may be feasible to stratify FXS or FXTAS patients for early intervention and improve clinical outcomes.

In this study we used an innovative approach called Deep-RACE, which combines RACE and next generation sequencing (Olivarius et al. 2009) to identify novel transcripts in high-throughput manner. This technique is highly sensitive and enables detection of very low abundance transcripts. Although our study uncovered two new lncRNAs, it is possible that we might have missed additional *FMR1*-derived transcripts because the RACE reaction is blocked by DNA sequences with high GC content, such as found in the promoter and 5′UTR of the *FMR1* gene. While there may well be other transcripts that are yet to be identified, this report of two novel lncRNAs, *FMR5* and *FMR6*, further highlights the complexity of the *FMR1* transcriptional landscape (Fig. 5). The functional properties of these lncRNAs remain to be explored. Should they prove to be functional, we may begin to see Fragile X Syndrome and its associated disorders as “single-locus” diseases in which multiple entities are affected by a repeat expansion in a single gene.

## Electronic supplementary material

Below is the link to the electronic supplementary material.

Supplementary material 1 (PDF 6413 kb)
