# Supplementary material for: Comprehensive analysis of the transcriptional landscape of the human FMR1 gene reveals two new long noncoding RNAs differentially expressed in Fragile X syndrome and Fragile X-associated tremor/ataxia syndrome
Source: Hum Genet. 2013 Sep 5;133(1):59–67. doi: 10.1007/s00439-013-1356-6 (PMC3898532; doi:10.1007/s00439-013-1356-6)
Supplement: Supplementary file 1 — Supplementary material 1 (PDF 6413 kb) [file 439_2013_1356_MOESM1_ESM.pdf]

SUPPLEMENTARY FIGURES AND LEGENDS

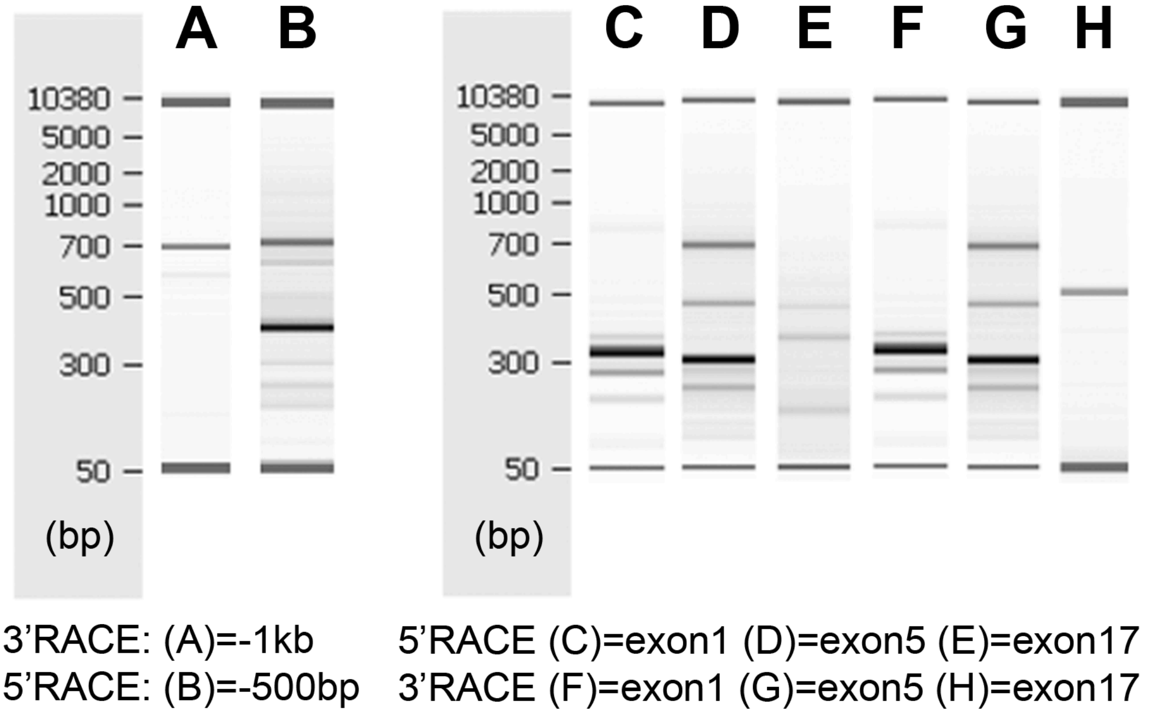

Online Resource 1. Visualization of 5’ and 3’RACE PCR products by capillary electrophoresis. 3’RACE (A) and 5’RACE (B), to detect sense-oriented transcripts, were performed 1kb and 0.5kb upstream of the *FMR1* transcription start site, respectively, and the PCR products were visualized by capillary electrophoresis. In order to detect antisense transcripts, 5’RACE (C-E) and 3’RACE (F-H) were performed on exon 1, exon 5 and exon 17 and the size of PCR products was detected with capillary electrophoresis.

FMR5

5’_(+1)GGTACTAAGTTCAATGCTGGCAGTCGTTTCTTCTTTTTTTTTCTTTTCTGTTTTGCTCA
CCGATTTCTCGTTAGCACTTAGCACAGTGTCTGGCACACGATAGATGCTCCGTCAACTTCT
CAGTTGGATACCAGCATCCCGAAGGGAACATGGATTAAGGCAGCTATAAGCACGGTGTAA
AAACAGGAATAAGAAAAAGTTGAGGTTTGTTTCACAGTGGAATGTAAAGGGTTGCAAGGAG
GTGCATCGGCCCCTGTGGACAGGACGCATGACTGCTACACACGTGTTCACCCCACCCTCT
GGCACAGGGTGCACATACAGTAGGGGCAGAAATGAACCTCAAGTGCTTAACACAATTTTTA
AAAAATATATAGTCAAGTGAAAGTATGAAAATGAGTTGAGGAAAGGCGAGTACGTGGGTCA
AAGCTGGGTCTGAGGAAAGGCTCACATTTTGAGATCCCGACTCAATCCATGTCCCTTAAAG
GGCACAGGGTGTCTCCACAGGGCCGCCCAAAATCTGGTGAGAGAGGGCGTAGACGCCTC
ACCTTCTGCCTCTACGGGTCACAAAAGCCTGGGTCACCCTGGTTGCCACTGTTCCTAGTTC
AAAGTCTTCTTCTGTCTAATCCTTCACCCCTATTCTCGCCTTCCACTCCACCTCCCGCTCAG
TCAGACTGCGCTACTTTGAACCGGACCAAACCAAACCAAACCAAACCAAACCAAACCAGAC
CAGACACCCCCTCCCGCGGAATCCCAGAGAGGCCGAACTGGGATAACCGGATGCATTTGA
TTTCCCACGCCACTGAGTGCACCTCTGCAGAAATGGGCGTTCTGGCCCTCGCGAGGCAGT
GCGACCTGTCACCGCCCTTCAGCCTTCCCGCCCTCCACCA**(+887)**

FMR6

5’_(+1)AGGGGGAGGAAGAGGACAAGGAGGAAGAGGACGTGGAGGAGGCTTCAAAGGAAA
CGACGATCACTCCCGAACAGATAATCGTCCACGTAATCCAAGAGAGGCTAAAGGAAGAAC
AACAGATGGATCCCTTCAGATCAGAGTTGACTGCAATAATGAAAGGAGTGTCCACACTAAA
ACATTACAGAATACCTCCAGTGAAGGTAGTCGGCTGCGCACGGGTAAAGATCGTAACCAG
AAGAAAGAGAAGCCAGACAGCGTGGATGGTCAGCAACCACTCGTGAATGGAGTACCCTAA
ACTGCATAATTCTGAAGTTATATTTCCTATACCATTTCCGTAATTCTTATTCCATATTAGAAAA
CTTTGTTAGGCCAAAGACAAATAGTAGGCAAGATGGCACAGGGCATGAAATGAACACAAAT
TATGCTAAGAATTTTTTATTTTTTGGTATTGGCCATAAGCAACAATTTTCAGATTTGCACAAA
AAGATACCTTAAAATTTGAAACATTGCTTTTAAAACTACTTAGCACTTCAGGGCAGATTTTAG
TTTTATTTTCTAAAGTACTGAGCAGTGATATTCTTTGTTAATTTGGACCATTTTCCTGCATTG
GGTGATCATTCACCAGTACATTCTCAGTTTTTCTTAATATA**(+650)_3’**

Online Resource 2. Sequence of *FMR5* and *FMR6*. Alignment of Deep-RACE reads to the human genome identifies *FMR5* and *FMR6.*

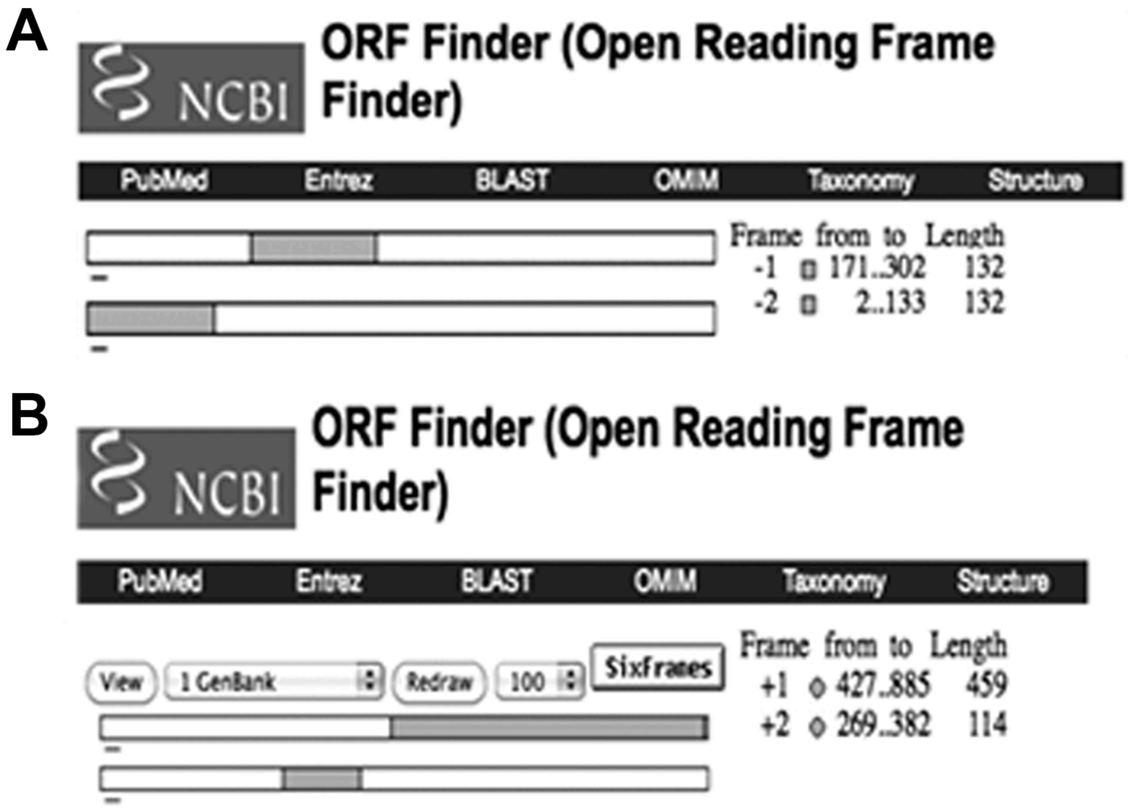

Online Resource 3. Interrogation of open reading frames (ORFs) in *FMR5* and *FMR6*. The entire sequence of FMR6 was interrogated for the presence of ORF using the NCBI's ORF Finder program. (A) Two ORFs of short length (132 nt each) were found in *FMR6*. (B) The partial sequence of *FMR5* contains two putative ORFs of 114 nt and 459 nt.

Online Resource 3. List of primers used in 5'RACE protocol.

**Name**	**Sequence**	**Application**
GSP1- Set 2	ACACCGTGCTTATAGCTGCC	5'RACE-sense RT
GSP2- Set 2	CACCTCCTTGCAACCCTTTA	5'RACE-sense PCR1
GSP3- Set 2	TAGCAGTCATGCGTCCTGTC	5'RACE-sense nested PCR
GSP1-Exon 17	AGTAGGCAAGATGGCACAGG	5'RACE-AS RT
GSP2-Exon 17	CACAGGGCATGAAATGAACA	5'RACE-AS PCR1
GSP3-Exon 17	GGGCATGAAATGAACACAAA	5'RACE-AS nested PCR
GSP1- Set 1	GTAGACGCCTCACCTTCTGC	5'RACE-AS RT
GSP2- Set 1	CTCACCTTCTGCCTCTACGG	5'RACE-AS PCR1
GSP3- Set 1	ACCAAACCAAACCAAACCAA	5'RACE-AS nested PCR
GSP1- Set 2	GGCAGCTATAAGCACGGTGT	5'RACE-AS RT
GSP2- Set 2	TAAAGGGTTGCAAGGAGGTG	5'RACE-AS PCR 1
GSP3- Set 2	GACAGGACGCATGACTGCTA	5'RACE-AS nested PCR

Online Resource 4. List of primers used in 3'RACE protocol.

Name	Sequence	Application
GSP1- Set 1	GTAGACGCCTCACCTTCTGC	3'RACE-sense PCR1
GSP2- Set 1	CTCACCTTCTTGCCTCTACGG	3'RACE-sense nested PCR
GSP1- Set 1	CACCTCCTTGCAACCCTTTA	3'RACE-AS PCR1
GSP2- Set 1	ACACCGTGCTTATAGCTGCC	3'RACE-AS nested PCR
GSP1- Set 2	CCGTAGAGGCAGAAGGTGAG	3'RACE-AS PCR1
GSP2- Set 2	GCAGAAGGTGAGGCGTCTAC	3'RACE-AS nested PCR
Exon 1	CACCAGCTCCTCCATCTTCT	3'RACE-AS PCR1
Exon 1-Nested	TTCTCTTCAGCCCTGCTAGC	3'RACE-AS nested PCR
Exon 5	CTTTTCTGGCAGGTTTGTTG	3'RACE-AS PCR1 & nested PCR
Exon 17	AGCCGACTACCTTCACTGGA	3'RACE-AS PCR1 & nested PCR

Online Resource 5. Description of brain tissue samples

Sample ID	Brain Region	Disease	Source	Age/Sex
1790	Occipital cortex	Control	NICHD	13/M
5391	Prefrontal cortex	Control	NICHD	8/M
5558	Prefrontal cortex	Control	NICHD	5/M
914	Prefrontal cortex	Control	NICHD	20/M
1024	Prefrontal cortex	Control	NICHD	14/M
1158	Prefrontal cortex	Control	NICHD	16/M
1347	Prefrontal cortex	Control	NICHD	19/F
1846	Prefrontal cortex	Control	NICHD	20/F
4669	Prefrontal cortex	Control	NICHD	16/M
5168	Prefrontal cortex	Control	NICHD	15/F
5173	Prefrontal cortex	Control	NICHD	10/F
4806	Occipital cortex	Full mutation	NICHD	9/M
5319	Occipital cortex	Full mutation	NICHD	71/M
1421	Frontal cortex	Full mutation	NICHD	69/M
1031-08GP	Cerebellum	Full mutation	MIND	57/M
1033-08WS	Cerebellum	Full mutation	MIND	74/M
1031-09LZ	Cerebellum	Full mutation	MIND	64/M
JS-03	Cerebellum	Full mutation	MIND	25/M
4664	Occipital cortex	Premutation	NICHD	71/M
4751	Occipital cortex	Premutation	NICHD	21/M
5212	Parieto-occipital cortex	Premutation	NICHD	80/M
4555	Occipital cortex	Premutation	NICHD	80/M
5006	Parietal cortex	Premutation	NICHD	85/M

Online Resource 6. List of primers used for quantitative RT-PCR.

Name	Sequence	Application
FMR1	Hs00924547_m1 (Applied Biosystem)	qPCR - Taqman
FMR4 RT	ATTGCTGGCAGTCGTTTCTT	FMR4 Strand-specific RT
FMR4 (FW)	ACCAAACCAAACCAAACCAA	qPCR - SYBR Green
FMR4 (REV)	GTGGGAAATCAAATGCATCC	qPCR - SYBR Green
FMR5	GTTATCCCAGTTCGGCCTCT	FMR5 Strand-specific RT
FMR5 (FW)	CGAGTACGTGGGTCAAAGCT	qPCR - Taqman
FMR5 (REV)	GACATGGATTGAGTCGGGATCT	qPCR - Taqman
FMR5 (Probe)	CAAAATGTGAGCCTTTCCTC	qPCR - Taqman
FMR6 (FW)	AGCACTTCAGGGCAGATTTT	qPCR - SYBR Green & FMR6 Strand-specific RT
FMR6 (REV)	TGGTGAATGATCACCCAATG	qPCR - SYBR Green
G6PD	Cf02646199_m1 (Applied Biosystem)	qPCR - Taqman
CYC	Cf03986523_gh (Applied Biosystem)	qPCR - Taqman
